# Supplementary material for: Magnetite-Arginine Nanoparticles as a Multifunctional Biomedical Tool
Source: Nanomaterials (Basel). 2020 Oct 13;10(10):2014. doi: 10.3390/nano10102014 (PMC7600042; doi:10.3390/nano10102014)
Supplement: Supplementary file 1 [file nanomaterials-10-02014-s001.pdf]

nanomaterials

Supporting Information

Magnetite-Arginine Nanoparticles as a Multifunctional Biomedical Tool

Victoria E. Reichel [1,2], Jasmin Matuszak [3,4], Klaas Bente [1,5], Tobias Heil [1], Alexander Kraupner [6], Silvio Dutz [7], Iwona Cicha [3] and Damien Faivre [1,8,*]

1 Max Planck Institute of Colloids and Interfaces, Department of Biomaterials, Am Mühlenberg 1, 14476 Potsdam, Germany; victoria.reichel@univ-paris-diderot.fr (V.E.R.); klaas.bente@bam.de (K.B.); tobias.heil@mpikg.mpg.de (T.H.)
2 Laboratoire "Matière et Systèmes Complexes" (MSC), UMR 7057 CNRS, Université Paris 7 Diderot, 75205 Paris Cedex 13, France
3 Section of Experimental Oncoclogy and Nanomedicine (SEON), ENT Department, University Hospital Erlangen, Friedrich-Alexander-Universität Erlangen-Nürnberg, Glückstraße 10a, 91054 Erlangen, Germany; jasmin.matuszak@charite.de (J.M.); iwona.cicha@uk-erlangen.de (I.C.)
4 Department of Anesthesiology, Kurume University Hospital, Cognitive and MolecularResearch Institute of Brain Diseases, Kurume University, 65-1, Asahimachi, Kurume 830-0011, Japan
5 Bundesanstalt für Materialforschung und -prüfung, Unter den Eichen 87, 12205 Berlin, Germany
6 nanoPET Pharma GmbH, Luisencarrée Robert-Koch-Platz 4, 10115 Berlin, Germany; alexander.kraupner@nanopet.de
7 Institute of Biomedical Engineering and Informatics, Technische Universität Ilmenau, PF 100565, 98684 Ilmenau, Germany; Silvio.Dutz@tu-ilmenau.de
8 Aix-Marseille Université, CEA, CNRS, BIAM, 13108 Saint Paul lez Durance, France
* Correspondence: damien.faivre@cea.fr

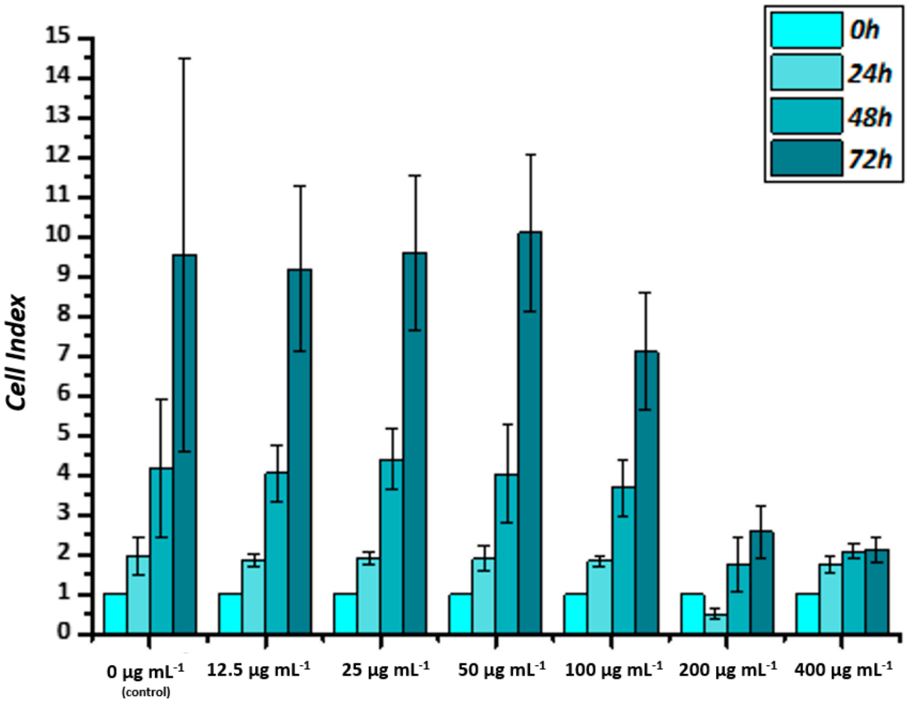

Figure S1. Biological effects of Resovist® particles on endothelial cells grown in static conditions by real-time cell analysis. HUVECs were seeded 24 h before nanoparticle application. After these initial 24 h, particles in different concentrations were added and cell index was monitored up to 72 h post application. Cell Index is displayed as x-fold of untreated controls. Data are expressed as mean ± SEM of n = 3 experiments.

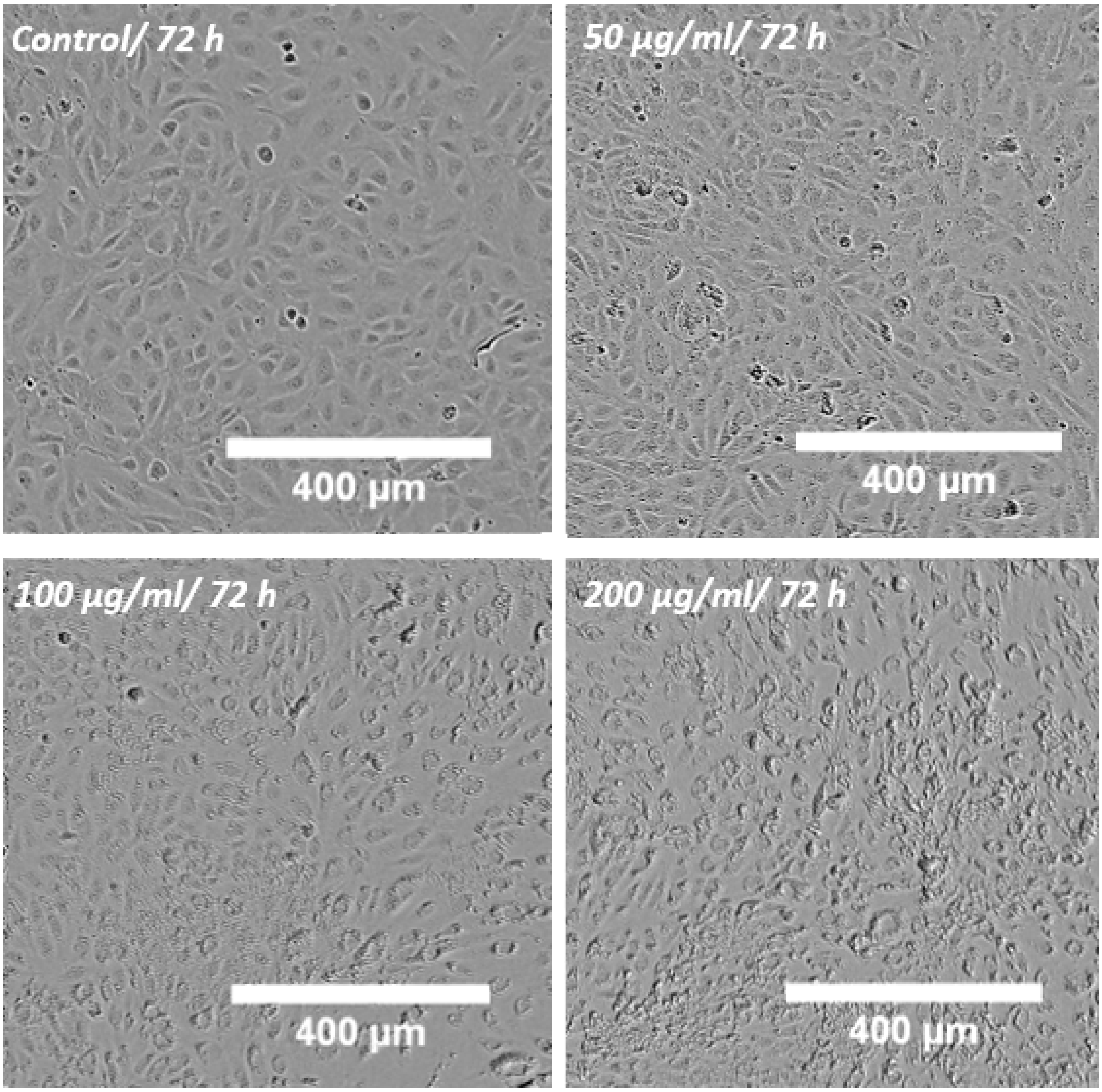

Figure S2. Live-cell microscopy of HUVECs treated with Resovist® particles: Phase contrast images of HUVECs were taken after 72 h post particle addition. (a) Untreated control HUVECs, (b) HUVECs treated with 50 µg mL^{-1}, (c) HUVECs treated with 100 µg mL^{-1} and (d) HUVECs treated with 200 µg mL^{-1} Resovist® particles.

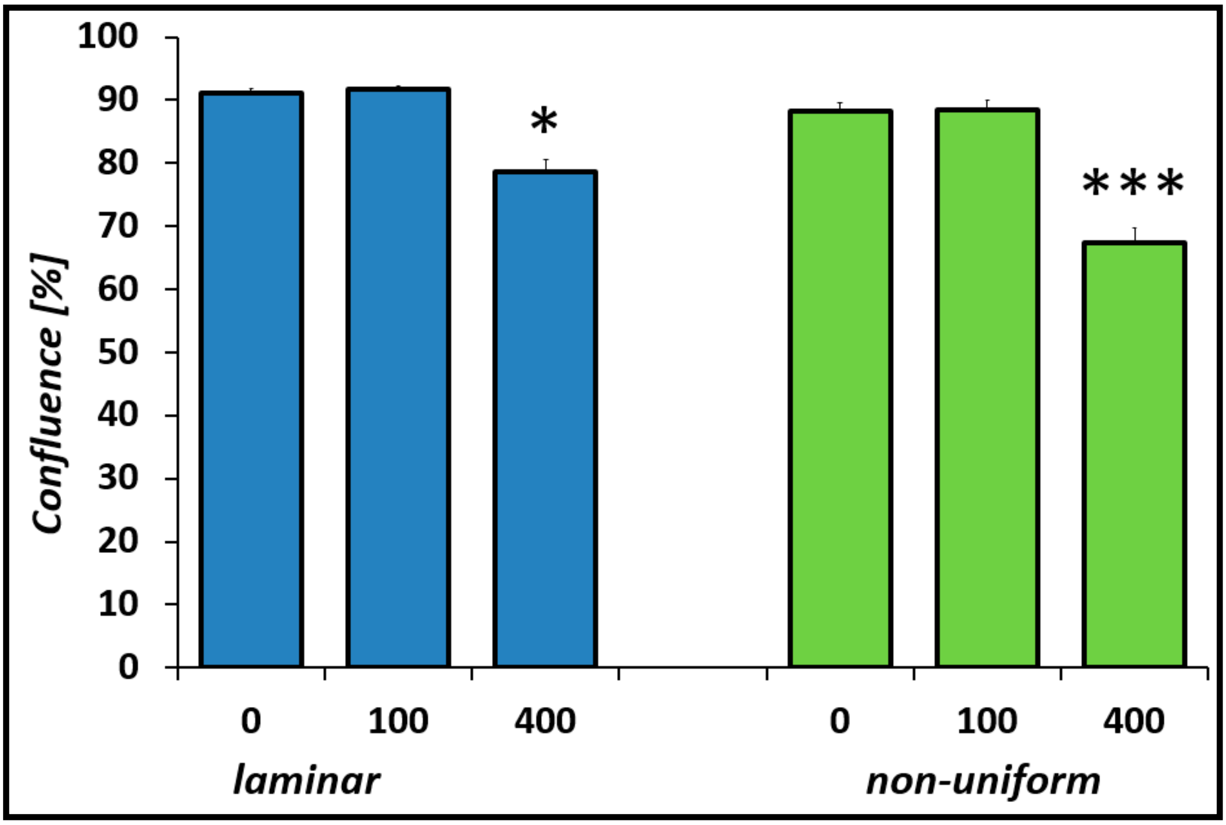

Figure S3. The graphs show the semi-quantitative analysis of confluence in non-uniform (green) and laminar (blue) region of the bifurcating channels according to the fluorescent images shown in Figure 4. The analysis was done using ImageJ software. n=3, Data are expressed as mean ± SEM, *$p<0.05$, ** $p<0.01$, ***$p<0.001$ vs. corresponding control (one-way Anova).
